# Complete Genome Sequence of the *Mesoplasma florum* W37 Strain

**DOI:** 10.1128/genomeA.00879-13

**Published:** 2013-11-27

**Authors:** Vincent Baby, Dominick Matteau, Thomas F. Knight, Sébastien Rodrigue

**Affiliations:** Département de Biologie, Université de Sherbrooke, Sherbrooke, Canadaa; Ginkgo Bioworks, Boston, Massachusetts, USAb

## Abstract

*Mesoplasma florum* is a small-genome fast-growing mollicute that is an attractive model for systems and synthetic genomics studies. We report the complete 825,824-bp genome sequence of a second representative of this species, *M. florum* strain W37, which contains 733 predicted open reading frames and 35 stable RNAs.

## GENOME ANNOUNCEMENT

*Mesoplasma florum*, first described as *Acholeplasma florum* in 1984 by McCoy and colleagues (1), is a mollicute that is closely related to mycoplasma but shows higher growth rates and has no known pathogenic potential and no sterol requirement. The complete genome sequence of a first representative, *M. florum* L1, was deposited in GenBank in 2004 (RefSeq NC_006055.1). Its small genome of <800 kbp positions this strain among some of the simplest free-living organisms. In addition, *M. florum* is closely related to mycoplasma species that have been used for whole-genome cloning (2–4) and genome transplantation (4, 5) experiments, suggesting that similar techniques could be adapted for engineering its genome. Here, we report the complete genome sequence of *M. florum* W37, which will allow comparative analysis within this species.

*M. florum* W37 was isolated from a *Solidago* sp. flower in 1982 near Gibson City, IL, by Robert F. Whitcomb (Beltsville Agricultural Research Center, Beltsville, MD) (6). The strain was obtained from the *Mollicutes* strain collection at Purdue University and grown at 34°C in ATCC 1161 medium to obtain sufficient DNA for sequencing. A first Illumina library was prepared from sheared DNA fragments of approximately 200 to 250 bp and sequenced with paired-end reads of 144 bp to assemble longer composite reads covering the entire insert (7). An additional Illumina library consisting of inserts of 600 ± 150 bp was sequenced with paired-end reads of 100 bp. Finally, a Pacific Biosciences RS library (using C_2_ chemistry) was generated with an insert of ≥5 kbp to obtain longer sequences that were error corrected using the Illumina sequences. All sequences were assembled using the Roche gsAssembler version 2.6 and Ray version 2.1.0 (8). The assemblies were merged and manually inspected before manual finishing with Sanger sequencing reads obtained either from PCR amplifications or directly from genomic DNA. The resulting chromosome of 825,824 bp, with a GC content of 26.95%, was submitted to the RAST genome annotation server (9). A total of 768 genes, including 733 open reading frames and 35 stable RNAs, were predicted. Of the protein-encoding genes, 37 are unique to *M. florum* W37 while 645 are shared with the *M. florum* L1 strain. Sequencing the genomes of additional *M. florum* strains will help define the core genome of this species and identify genes that could potentially be deleted toward the creation of a genome-reduced strain.

### Nucleotide sequence accession number.

The completed genome sequence of *M. florum* W37 was deposited in GenBank under accession number CP006778.
